# Examination Stress Results in Attentional Bias and Altered Neural Reactivity in Test-Anxious Individuals

**DOI:** 10.1155/2018/3281040

**Published:** 2018-03-20

**Authors:** Xiaocong Zhang, Yunying Dong, Renlai Zhou

**Affiliations:** ^1^Research Center for Learning Science, Southeast University, Nanjing 210096, China; ^2^School of Psychology, Nanjing University of Chinese Medicine, Nanjing 210023, China; ^3^School of Education, Jiangsu University of Technology, Changzhou 213001, China; ^4^Department of Psychology, School of Social and Behavioral Sciences, Nanjing University, Nanjing 210023, China; ^5^National Key Laboratory of Cognitive Neuroscience and Learning, Beijing Normal University, Beijing 100875, China

## Abstract

Examination stress occurs so frequently in the lives of students. The neural mechanisms of attentional bias induced by examination stress in test-anxious individuals remain unclear. Accordingly, we investigated the attentional bias toward test-related threatening words in selected high and low test-anxious participants under the stress of final examinations by using an event-related potential (ERP) technique. A classic dot-probe paradigm was adopted with a test-related/test-unrelated threatening word and a neutral word pair as cues. Results showed attention bias and enhanced N200 amplitude toward test-related threat in high test-anxious individuals, whereas avoidance of test-related threat and decreased N200 amplitude were shown in low test-anxious individuals. Additionally, ERP data revealed the relatively enhanced LPP amplitude in low test-anxious participants compared with that in high test-anxious participants. No attentional bias toward test-unrelated threat was found. In conclusion, examination stress resulted in attentional bias and functional perturbations of a brain circuitry that reacted rapidly to test-related threat in high test-anxious individuals.

## 1. Introduction

Examinations occur so frequently and assume so much importance in the lives of students. College students report that examinations are the main sources of considerable stress [1]. Routine experiences of examination stress can lead to adverse psychological and physiological health [2, 3]. It has been estimated that about 15% to 20% of college students suffer from test anxiety [4]. Test anxiety is characterized by feelings of tension, worrisome thoughts, and the activation of the autonomic nervous system when an individual faces evaluative achievement-demanding situations [5]. Test anxiety can magnify stressful experiences about an examination situation. High test-anxious (HTA) individuals produce significantly higher physiological responses during examination settings than low test-anxious (LTA) individuals, and larger increases in physiological arousal are often associated with poorer exam or task performance [6].

The issue of how test-anxious individuals process threat (especially test-related threat) under examination stress is of particular interest because of the important implications of developing coping strategies and effective treatments for students [7, 8]. Previous studies showed that the attention system of anxious individuals might be abnormally sensitive to threat-related stimuli in the environment [9]. This attention pattern has been implicated in the etiology and maintenance of anxiety disorders [10]. As far as we know, only a few studies have investigated the attentional bias of test-anxious individuals. The existing studies suggested that test-anxious individuals show attentional bias toward test-related threatening information under examination stress [11–13]. However, these studies have not examined test-anxious individuals' attentional bias toward test-unrelated threatening stimuli. This issue has critical implications for providing the impetus for interventions through attentional bias modification training. Recently, researchers have tried to use computerized attention-training tasks to modify attentional bias patterns of test-anxious participants and revealed significant reduction in anxiety vulnerability [7]. The attentional bias pattern needs to be systematically investigated before more effective interventions are carried out. If test-anxious individuals also show attentional bias toward test-unrelated threat, attention training should be carried out to help them modify the attentional bias pattern.

Furthermore, no research has investigated the neural mechanisms of attentional bias induced by examination stress in test-anxious subjects. The existing studies relying on reaction times (RTs) and attentional bias scores (ABS) are insufficient to identify the underlying neural correlates of attentional processing and their timing. In contrast to RTs and ABS, which reflect the combined effects of a sequence of many distinct neural processes, the ERP technique can obtain an online measure of attentional processing and show how the allocation of attention unfolds over the course of a trial [14]. The present research investigated neural mechanisms of attentional bias in test-anxious subjects, by using the ERP technique in conjunction with the traditional RT measure. ERP studies have revealed an initial shift of attention toward threat in trait-anxious individuals, as measured with the larger amplitudes for the P1 [15, 16] and/or N200 component [17], and sustained engagement with threat over time, as measured with the late positive potential (LPP) [18, 19]. Augmentation of the P1 component was found among high trait-anxious individuals, which was attributed to greater attention allocation to the threatening relative to neutral stimuli [15]. The N200 component has been well validated and was used to examine the allocation of attention to emotional stimuli [17, 20]. LPP was also used to investigate whether a threatening stimulus elicits sustained engagement. If attention was initially shifted to threatening stimuli (reflected by P1 or N200) but not maintained on them, threat-related modulation of the LPP would likely not to be observed.

The present study employed the classic dot-probe task to investigate the attentional bias of test-anxious individuals. Attentional bias was inferred from different RTs toward probes that replaced threatening stimuli (i.e., test-related and test-unrelated words) compared to probes that replaced neutral stimuli [21]. If a test-anxious individual's attention was abnormally sensitive to a threatening stimulus, RTs would be shorter for probes that replaced threatening stimuli compared to RTs for probes that replaced neutral stimuli. The research question is as follows: Do HTA individuals undergoing examination stress, compared with LTA individuals, show attentional bias and altered neural reactivity toward test-related threat rather than test-unrelated threat (test-unrelated threatening words)? Test-anxious individuals were sensitive to environmental stimuli that are relevant to their specific anxiety-related schemas [5]. That is, HTA participants' attention should be oriented toward the location and direction of test-related threats in examination settings [12]. Therefore, we expected to see stronger evidence of attentional bias toward test-related threat rather than toward test-unrelated threat in the HTA group. More specifically, this pattern of results would be typified in both behavioral and electrophysiological data. In the behavioral data, this prediction would be achieved if test-anxious individuals show faster RTs or positive attention bias scores for test-related threat. In the ERP data, this prediction would be supported by the finding of a larger amplitude for the P1 and/or N200 components in HTA relative to LTA individuals in response to cued test-related threatening words. In a word, we expected to further illuminate the association between examination stress, attentional bias, and brain activation by using the classic dot-probe task.

## 2. Materials and Methods

### 2.1. Participants

Forty-five volunteers were selected from a larger pool of undergraduate students who had completed the short form of the Test Anxiety Inventory [22] in Chinese (short form of the TAI-C) [23]. HTA participants (*N* = 22; mean age, 19.86 ± 1.28 years; 11 females) were defined as those scoring more than 13 (*M* = 16.09, SD = 1.87); LTA participants (*N* = 23; mean age, 19.67 ± 1.27 years; 9 females) were those scoring less than 8 (*M* = 6.04, SD = .88) in our study. These cut-off points represented approximately the upper and lower 16% of preliminary norms of the short form of the TAI-C (one standard deviation above and below the mean) [20]. The groups differed in TAI-C, *t*(43) = 22.85, *p* < .001. All participants signed the written informed consent and had self-reported normal or corrected-to-normal eyesight.

### 2.2. Stimuli and Task

Stimuli consisted of 72 test-related threatening words (TR) and 72 test-unrelated threatening words (TU), individually matched with 144 neutral words (*N*). Word pairs were presented one above the other in Song font size 21 and were 3 cm apart. Whether a threatening or neutral word was presented at the top was randomly chosen.

The words were drawn from the Test Anxiety Word System [24], which provides a standardized set of emotional stimuli with normative ratings of threat dimension, test-related dimension, and familiar dimension. In the threat dimension, the TR set and the TU set did not significantly differ between each other, but both differed from the *N* set significantly (TR = 5.13 ± .37, TU = 5.18 ± .31, *N* = 2.65 ± .16; *F*_(2,285)_ = 3290.07, *p* < .001, *η*^2^_p_ = .96). In the test-related dimension, the TU set and the *N* set did not significantly differ between each other, but both differed from the TR set significantly (TR = 5.54 ± .34, TU = 2.66 ± .24, *N* = 2.64 ± .15; *F*_(2,285)_ = 4160.76, *p* < .001, *η*^2^_p_ = .97). In the familiar dimension, the three sets did not significantly differ between each other (TR = 5.51 ± .60, TU = 5.55 ± .57, *N* = 5.40 ± .48; *F*_(2,285)_ = 2.19, *p* = .11, *η*^2^_p_ = .02).

**Figure 1:**
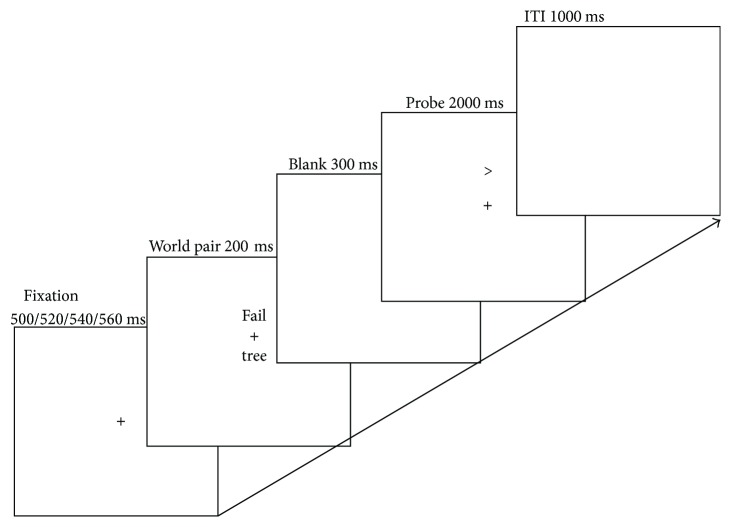
Example trial sequence in the dot-probe task.

In the dot-probe task (see Figure 1), each trial began with a central fixation cross, presented for 500/520/540/560 ms, followed by a word pair (i.e., cue) displayed for 200 ms. Immediately (300 ms) following the word pair, the probe stimulus (either left or right arrows) was randomly presented with equal regularity in the location of the centre of one of the words for 200 ms. Then, a blank screen was presented until a response was made or until 2000 ms had elapsed. Participants had to determine the orientation of the probes by pressing one of two prespecified buttons. Participants were instructed to respond as quickly and accurately as possible. The intertrial interval was 1000 ms. The task was split into four experimental blocks; each of the blocks contained 72 trials. The practice block including 12 trials was shown prior to the experimental blocks. The trials were presented in a new random order for each participant.

### 2.3. EEG Recording and Artifact Scoring

Electroencephalograms were recorded using a 256-channel system (Electrical Geodesics (EGI), USA). All electrode impedances were kept below 40 kΩ. All channels were referenced to the Cz channel, and data were collected using a 0.1–100 Hz bandpass filter. Signals were collected at 500 samples per second and digitized with a 16-bit A/D converter.

Trials with incorrect responses were eliminated from analysis, as well as trials containing eye blinks or artifacts exceeding ±70 *μ*V. Prior to data analyses, all ERP waveforms were low-pass filtered at 30 Hz, using a zero-phase shift digital filter [15]. Mean cue-evoked ERP amplitudes were epoched from −200 ms to 800 ms after the word pair onset, and probe-evoked ERP amplitudes were epoched from −200 ms to 500 ms after the probe onset [18].

### 2.4. Procedure

To investigate the neural reactivity induced by examination stress, participants carried out the procedures 1 week before their final examination. All participants were seated in a comfortable chair 80 cm from the computer screen in a sound-attenuated room. Instructions for the task were presented. Participants completed the practice block and the dot-probe task. Between blocks, several minutes of rest were taken appropriately. The EEG was recorded throughout the experiment. Finally, participants were thanked, paid, and debriefed after participation.

### 2.5. Data Analyses

#### 2.5.1. Behavioral Reaction Times

Erroneous responses were excluded from statistical analyses. RTs shorter than 200 ms or longer than 1500 ms were removed. Furthermore, RTs deviating more than 3 SDs from the individual mean RTs were excluded. Statistical analyses were run on 98% of the data. RTs were subjected to a 2 (groups: HTA and LTA) × 2 (word types: TR and TU) × 2 (congruency: congruent and incongruent) mixed-design ANOVA. Congruent corresponds to the probes replacing threatening words, while incongruent corresponds to the probes replacing neutral words. All variables were within subjects except for group. If the higher-order interactions including congruency were significant, an attentional bias index would be calculated (see below).

For each of the types of threatening words (TR and TU), the bias score was calculated following MacLeod et al. [25]. Bias score = 0.5 × (RT_incongruent_ − RT_congruent_), where incongruent corresponds to the probes replacing neutral words and congruent corresponds to the probes replacing threat words. Positive values reflect attention toward the threatening words, and negative values reflect attention away from the threatening words. A value of zero implies no attentional bias. Bias scores were analyzed using a 2 (groups: HTA and LTA) × 2 (word types: TR and TU) mixed-design ANOVA with the between-subjects factor group and the within-subjects factor word type.

#### 2.5.2. ERP Analysis

Based on previous reports in the literature [14, 15, 18, 26] and inspection of the grand mean ERPs, ERP analyses focused on the mean amplitudes of the P1 elicited by the word pairs (i.e., the cue) and the N200 and LPP evoked by the probe. Attention allocation was known to modulate P1 over occipital electrode sites. Thus, P1 was analyzed over occipital electrodes (O1 and O2) [15]. The N200 was analyzed at posterior electrode sites (P7 and P8), where N200 was typically maximal [14]. The LPP was analyzed at POz as it was typically maximal at posterior and parietal sites [18].

The P1 mean amplitude was computed between 120 and 140 ms after the presentation of word pairs and analyzed by repeated-measures analysis of variance (ANOVA), with group (HTA and LTA) as between-subjects factor and word type (TR and TU) and electrodes (O1 and O2) as within-subjects factors. The N200 peak amplitude was measured within the latency window of 180–200 ms and analyzed by repeated-measures analysis of variance (ANOVA), with group (HTA and LTA) as between-subjects factor and word type (TR and TU), congruency (congruent and incongruent), and electrodes (P7 and P8) as within-subjects factors. Finally, the LPP was scored as the mean activity between 300 and 500 ms at the electrode site POz and analyzed by repeated-measures analysis of variance (ANOVA), with group (HTA and LTA) as between-subjects factor and word type (TR and TU) and congruency (congruent and incongruent) as within-subjects factors. Planned comparisons were run to compare effects within the HTA and LTA groups. The Greenhouse-Geisser correction was used.

## 3. Results

### 3.1. Reaction Time Data

The 2 × 2 × 2 ANOVA on RTs revealed a significant interaction of group × word type × congruency, *F*_(1,43)_ = 8.656, *p* = .005, *η*^2^_p_ = .168.  Table 1 shows the RTs for the three-way interaction effect.

**Figure 2:**
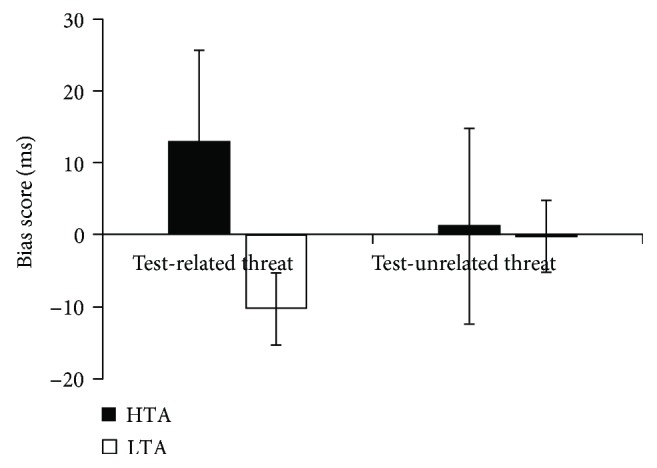
Bias scores to test-related (TR) and test-unrelated (TU) threat for HTA and LTA participants.

The 2 × 2 ANOVA on bias scores revealed a significant main effect of group, *F*_(1,43)_ = 18.799, *p* < .001, *η*^2^_p_ = .304 and a significant group × word type interaction, *F*_(1,43)_ = 8.656, *p* = .005, *η*^2^_p_ = .168 (see Table 2). Follow-up tests revealed that HTA individuals had a larger bias score for TR words compared with the LTA individuals, *F*_(1,43)_ = 23.469, *p* < .001, *η*^2^_p_ = .353. There was no significant difference between HTA and LTA individuals for the bias scores on TU words, *F*_(1,43)_ = .095, *p* = .760, *η*^2^_p_ = .002. As predicted, a comparison against zero revealed that HTA individuals had a positive bias score, *t*(21) = 3.20, *p* = .004, and LTA individuals had a negative bias score, *t*(22) = −3.88, *p* = .001, both for TR words (see Figure 2).

### 3.2. Analyses of Cue-Evoked ERPs

No significant effects were found for the P1 component time-locked to cue onset.

### 3.3. Analyses of Probe-Evoked ERPs

**Figure 3:**
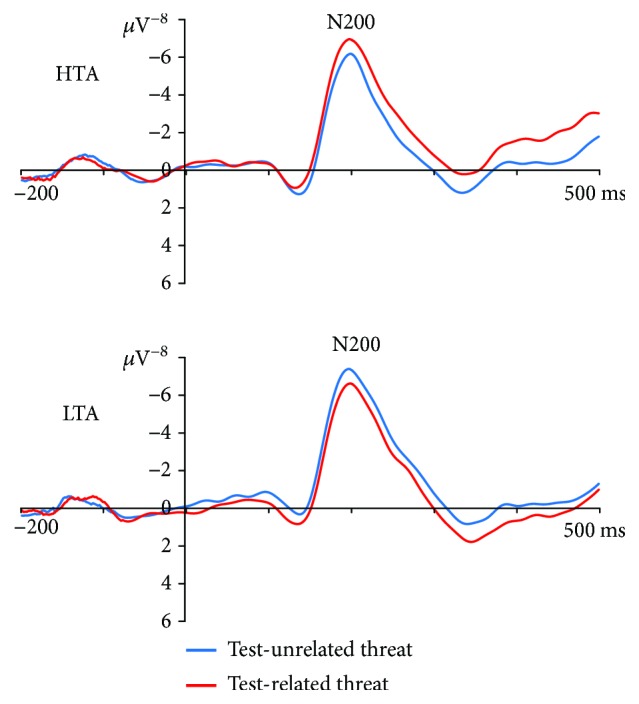
Grand-averaged ERPs evoked by probes in the incongruent test-unrelated (TU) condition and test-related (TR) condition at the P7 electrode site for HTA and LTA participants.

For the peak amplitude of the N200 component, the interaction of group × word type × congruency × electrodes was significant, *F*_(1,43)_ = 10.16, *p* = .003, *η*^2^_p_ = .19. Planned comparisons conducted on each group showed that for the HTA group, the amplitudes at the electrode site P7 in the incongruent TR-N condition (*M* = −6.96, SE = .72) are marginally more negative than those in the incongruent TU-N condition (*M* = −6.19, SE = .71), *p* = .051, while for the LTA group, the amplitudes at the electrode site P7 in the incongruent TR-N condition (*M* = −6.63, SE = .70) are significantly less negative than those in the incongruent TU-N condition (*M* = −7.40, SE = .69), *p* = .047 (see Figure 3). None of the other comparisons resulted in significant differences in N200 amplitude between the word pairs' conditions.

**Figure 4:**
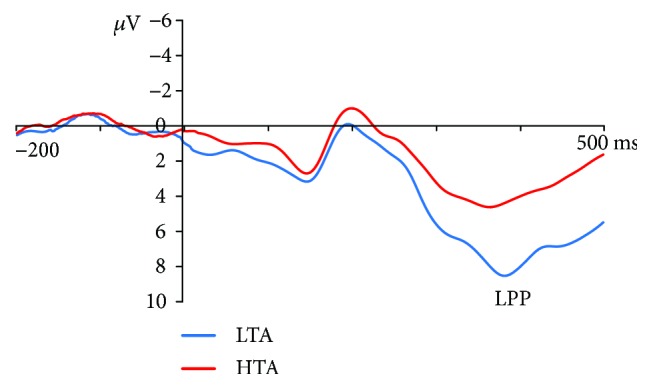
Grand-averaged late positive potential (LPP) evoked by probes at the POz electrode site for HTA (red) and LTA (blue) participants.

Grand-averaged ERP waveforms time-locked to the onset of the probe at the electrode site POz are presented in Figure 4. There was a significant main effect of group on the LPP such that the LTA group (*M* = 6.98, SE = 1.10) elicited a larger LPP compared with the HTA group (*M* = 3.57, SE = 1.12), *F*_(1,43)_ = 4.72, *p* = .04, *η*^2^_p_ = .10, and a significant word type × congruency interaction, *F*_(1,43)_ = 4.15, *p* = .048, *η*^2^_p_ = .09. Follow-up analyses revealed that for the congruent condition, a significantly higher LPP amplitude was elicited by the TU-N word pairs (*M* = 5.86, SE = .82) relative to the TR-N word pairs (*M* = 4.84, SE = .80), *F*_(1,43)_ = 5.51, *p* = .02, *η*^2^_p_ = .11. For incongruent condition, the LPP amplitude elicited by the TU-N word pairs (*M* = 5.29, SE = .86) did not, however, differ from that elicited by the TR-N word pairs (*M* = 5.10, SE = .85), *F*_(1,43)_ = .18, *p* = .67, *η*^2^_p_ = .004. No other main or interaction effects emerged from this analysis, *F*s < 2.29, *p*s > .08, *η*^2^_p_s < .06.

## 4. Discussion

The present study found that HTA participants undergoing the stress of academic examinations showed attentional bias toward TR with enhanced N200 amplitude, while LTA participants showed avoidance of them with decreased N200 amplitude. In addition, the electrophysiological data revealed no threat-related modulation of the LPP but relatively enhanced LPP amplitude in low test-anxious participants compared with that in high test-anxious participants.

The behavioral results of this study confirmed our predictions. In accord with the extant literature on attentional bias in test-anxious subjects [12, 13], this study provided evidence that test anxiety was characterized by an attentional bias toward test-related threatening stimuli under examination stress. Test anxiety predisposes HTA individuals to be susceptible to distraction and interference of test-related threat [5]. HTA individuals tend to consume a disproportionate amount of cognitive resources to scan the test environment for possible signs of test-related threat. This can help explain why HTA individuals are often associated with poorer exam or task performance under examination stress [27]. This study further clarified that test-anxious individuals did not show attentional bias toward test-unrelated threat. It confirmed the notion that test anxiety was a situation-specific form of trait anxiety and was characterized by intrusive anxiety-related behaviors and cognitions elicited by testing stimuli in academic or evaluative settings [5]. More effective interventions for test anxiety (e.g., attentional bias modification training [7]) should be carried out to help HTA individuals modify attentional bias patterns toward test-related threat.

In the present study, ERP results revealed that HTA individuals showed attentional bias toward TR with enhanced N200 amplitude and LTA individuals showed avoidance toward TR with decreased N200 amplitude. In previous studies of anxiety, N200 was used to examine the allocation of attention to emotional stimuli [14]. A larger N200 amplitude was associated with processing emotional than neutral stimuli in dot-probe task [17, 18]. The ERP results directly indicated that HTA individuals can quickly detect the threatening stimulus within about 200 ms after the emergence. We believed that N200 was likely to be consistent with the behavioral results, reflecting a significant initial shift of attention to test-related threat.

However, the electrophysiological data revealed no threat-related modulation of the LPP, which indicated that after initially shifting to threatening stimuli, HTA individuals did not show sustained attention to them [28]. This result was consistent with previous eye-tracking research which suggested that HTA individuals were characterized by initially attending to test-related threat but avoiding such stimuli later on [13]. Some ERP researches also found no evidence of sustained engagement with the threat-related stimuli in high trait-anxious participants [14, 18]. It meant that in order to reduce increased internal distress, highly anxious subjects may try to counterbalance their initial attention to threat by quickly disengaging attention away from the threat [29].

The present study also found that compared with LTA individuals, HTA individuals elicited decreased LPP amplitude. LPP is susceptible to top-down processing influences, and its magnitude can be modulated by psychological resources [30]. This result indicated that HTA individuals may have less cognitive resources to complete the dot-probe task. The attentional control theory proposes that anxiety affects the efficiency of the inhibition function, which can prevent attention being directed to task-irrelevant stimuli (e.g., test-related threat) [31]. As a consequence, HTA individuals may consume too much cognitive resources for task-irrelevant stimuli.

The present study failed to find threat-related ERP differences in the P1 component locked to the cue-processing phase of the dot-probe task. Previous studies were inconsistent in their findings on such modulation. Augmentation of the P1 component was found among anxious individuals [15], while a reduction in this component was also shown in anxious individuals [26]. Hence, although the behavioral results in the present study indicated that HTA individuals were selectively biased toward test-related threat, this was not mirrored by selective modulation of the P1 component to the cue display. This null result might be due to no difference in the threat level between test-related and test-unrelated threatening words in the classic dot-probe used here. The current study did not provide a definitive answer regarding threat- and anxiety-related modulations in the P1 component, leaving this issue open for future researches.

A noteworthy limitation of the present study is that although a facilitated response to probes that appear at the location of a test-related threat is interpreted as vigilance for threat, results can also be interpreted as a difficulty to disengage from the threat [32]. These data suggest that it is time to modify the dot-probe task (e.g., adding N-N trials) and to index the components of attentional bias toward test-related threat.

## 5. Conclusion

In summary, the present study demonstrated that examination stress resulted in attentional bias and functional perturbations of a brain circuitry that reacted rapidly to test-related threat in high test-anxious individuals.

## Figures and Tables

**Table 1:** Mean reaction times (in ms) in the dot-probe task for HTA and LTA participants (standard deviations in parentheses).

	Test-related threatening word		Test-unrelated threatening word	
	Incongruent	Congruent	Incongruent	Congruent
HTA group (*n* = 22)	621.13 (231.43)	595.16 (208.05)	602.03 (214.92)	599.59 (215.09)
LTA group (*n* = 23)	488.77 (150.79)	509.32 (155.56)	500.59 (153.32)	500.96 (157.67)

**Table 2:** Bias scores in the dot-probe task for HTA and LTA participants.

	Test-related threatening word		Test-unrelated threatening word	
	Mean (SD)	*t*-test^a^	Mean (SD)	*t*-test^a^
HTA group (*n* = 22)	12.98 (19.02)	3.20^∗^	1.22 (16.83)	.34
LTA group (*n* = 23)	−10.27 (12.70)	−3.88^∗^	−.18 (13.59)	−.07

^a^Results from a one-sample t-test between bias scores and zero (^∗^*p* < .05).

## Abbreviations

ABS: Attentional bias scores
HTA: High test-anxious
LTA: Low test-anxious
LPP: Late positive potential
RTs: Reaction times
TR: Test-related threatening words
TU: Test-unrelated threatening words.

## References

[1] Conley K. M., Lehman B. J. (2012). Test anxiety and cardiovascular responses to daily academic stressors. *Stress and Health*.

[2] Clutter L. B., Potter W. T., Alarbi A., Caruso J. F. (2017). Test anxiety and salivary cortisol levels in nursing students. *Nurse Educator*.

[3] Loft P., Thomas M. G., Petrie K. J., Booth R. J., Miles J., Vedhara K. (2007). Examination stress results in altered cardiovascular responses to acute challenge and lower cortisol. *Psychoneuroendocrinology*.

[4] Ergene T. (2003). Effective interventions on test anxiety reduction. *School Psychology International*.

[5] Spielberger C. D., Vagg P. R., Speilberger C. D., Vagg P. R. (1995). Test anxiety: a transactional process model. *Test Anxiety: Theory, Assessment and Treatment*.

[6] Jiang Q., Zhao Y., Hou Q., Sui H., Lv H., Liu Z. (2016). Test anxiety correlates with 24-hour ambulatory blood pressure and angiotensin II in high school students. *Journal of Hypertension*.

[7] Cai W. P., Pan Y., Chai H. (2018). Attentional bias modification in reducing test anxiety vulnerability: a randomized controlled trial. *BMC Psychiatry*.

[8] de Hullu E., Sportel B. E., Nauta M. H., de Jong P. J. (2017). Cognitive bias modification and CBT as early interventions for adolescent social and test anxiety: two-year follow-up of a randomized controlled trial. *Journal of Behavior Therapy and Experimental Psychiatry*.

[9] Barry T. J., Vervliet B., Hermans D. (2015). An integrative review of attention biases and their contribution to treatment for anxiety disorders. *Frontiers in Psychology*.

[10] Kuckertz J. M., Amir N. (2015). Attention bias modification for anxiety and phobias: current status and future directions. *Current Psychiatry Reports*.

[11] Keogh E., French C. C. (2001). Test anxiety, evaluative stress, and susceptibility to distraction from threat. *European Journal of Personality*.

[12] Putwain D. W., Langdale H. C., Woods K. A., Nicholson L. J. (2011). Developing and piloting a dot-probe measure of attentional bias for test anxiety. *Learning and Individual Differences*.

[13] Dong Y. Y., Beuckelaer A. D., Yu L., Zhou R. L. (2016). Eye-movement evidence of the time-course of attentional bias for threatening pictures in test-anxious students. *Cognition and Emotion*.

[14] Kappenman E. S., Farrens J. L., Luck S. J., Proudfit G. H. (2014). Behavioral and ERP measures of attentional bias to threat in the dot-probe task: poor reliability and lack of correlation with anxiety. *Frontiers in Psychology*.

[15] Eldar S., Yankelevitch R., Lamy D., Bar-Haim Y. (2010). Enhanced neural reactivity and selective attention to threat in anxiety. *Biological Psychology*.

[16] Rossignol M., Campanella S., Bissot C., Philippot P. (2013). Fear of negative evaluation and attentional bias for facial expressions: an event-related study. *Brain and Cognition*.

[17] Sass S. M., Heller W., Fisher J. E. (2014). Electrophysiological evidence of the time course of attentional bias in non-patients reporting symptoms of depression with and without co-occurring anxiety. *Frontiers in Psychology*.

[18] Kappenman E. S., MacNamara A., Proudfit G. H. (2015). Electrocortical evidence for rapid allocation of attention to threat in the dot-probe task. *Scan*.

[19] Schupp H. T., Ohman A., Junghofer M., Weike A. I., Stockburger J., Hamm A. O. (2004). The facilitated processing of threatening faces: an ERP analysis. *Emotion*.

[20] Holmes A., Mogg K., Fockert J., Nielsen M., Bradley B. (2014). Electrophysiological evidence for greater attention to threat when cognitive control resources are depleted. *Cognitive, Affective and Behavioral Neuroscience*.

[21] Rooijen R.v., Ploeger A., Kret M. (2017). The dot-probe task to measure emotional attention: a suitable measure in comparative studies?. *Psychonomic Bulletin & Review*.

[22] Taylor J., Deane F. P. (2002). Development of a short form of the Test Anxiety Inventory (TAI). *The Journal of General Psychology*.

[23] Dong Y. Y., Zhou R. L., Gao X., Jiao F., Guo W. (2011). Reliability and validity of the Chinese version of Test Anxiety Inventory (TAI) Short Form in college students. *Chinese Mental Health Journal*.

[24] Gao X., Zhou R. L. (2013). On the inhibition of selective attention by people with test anxiety. *Chinese Journal of Special Education*.

[25] MacLeod C., Mathews A., Tata P. (1986). Attentional bias in emotional disorders. *Journal of Abnormal Psychology*.

[26] Mueller E. M., Hofmann S. G., Santesso D. L., Meuret A. E., Bitran S., Pizzagalli D. A. (2009). Electrophysiological evidence of attentional biases in social anxiety disorder. *Psychological Medicine*.

[27] Shi Z., Gao X., Zhou R. L. (2014). Emotional working memory capacity in test anxiety. *Learning and Individual Differences*.

[28] Olofsson J. K., Nordin S., Sequeira H., Polich J. (2008). Affective picture processing: an integrative review of ERP findings. *Biological Psychology*.

[29] Calvo M. G., Avero P. (2005). Time course of attentional bias to emotional scenes in anxiety: gaze direction and duration. *Cognition and Emotion*.

[30] Núñez-Peña M. I., Suárez-Pellicion M. (2015). Processing of multi-digit additions in high math-anxious individuals: psychophysiological evidence. *Frontiers in Psychology*.

[31] Eysenck M. W., Derakshan N. (2011). New perspectives in attentional control theory. *Personality and Individual Differences*.

[32] Koster E. H. W., Crombez G., Verschuere B., De Houwer J. (2004). Selective attention to threat in the dot probe paradigm: differentiating vigilance and difficulty to disengage. *Behaviour Research and Therapy*.

